# The characteristics of the frequent exacerbator with chronic bronchitis phenotype and non-exacerbator phenotype in patients with chronic obstructive pulmonary disease: a meta-analysis and system review

**DOI:** 10.1186/s12890-020-1126-x

**Published:** 2020-04-23

**Authors:** Jian-jun Wu, Hong-ri Xu, Ying-xue Zhang, Yi-xuan Li, Hui-yong Yu, Liang-duo Jiang, Cheng-xiang Wang, Mei Han

**Affiliations:** 1 The Third Affiliated Hospital of Beijing University of Chinese Medicine, No. 51, Xiaoguan Street outside Anding Men, Chaoyang, Beijing, 100029 People’s Republic of China; 2 Dongzhimen Hospital, Beijing University of Chinese Medicine, No.5 Haiyuncang, Dongcheng District, Beijing, 100700 People’s Republic of China; 3 Centre for Evidence-Based Chinese Medicine, Beijing University of Chinese Medicine, 11 East Road North 3rd Ring Road, Beijing, 100029 People’s Republic of China

**Keywords:** FE-CB, NE, Phenotype, COPD, Pulmonary function, Meta-analysis

## Abstract

**Background:**

Chronic obstructive pulmonary disease (COPD) patients with different phenotypes show different clinical characteristics. Therefore, we conducted a meta-analysis to explore the clinical characteristics between the non-exacerbator (NE) phenotype and the frequent exacerbator with chronic bronchitis (FE-CB) phenotype among patients with COPD.

**Methods:**

CNKI, Wan fang, Chongqing VIP, China Biology Medicine disc, PubMed, Cochrane Library, and EMBASE databases were searched from the times of their inception to April 30, 2019. All studies that reported the clinical characteristics of the COPD phenotypes and which met the inclusion criteria were included. The quality assessment was analyzed by Cross-Sectional/Prevalence Study Quality recommendations. The meta-analysis was carried out using RevMan5.3.

**Results:**

Ten cross-sectional observation studies (*n* = 8848) were included. Compared with the NE phenotype, patients with the FE-CB phenotype showed significantly lower forced expiratory volume in 1 s percent predicted (FEV_1_%pred) (mean difference (MD) -8.50, 95% CI -11.36–-5.65, *P* < 0.001, *I*^*2*^ = 91%), forced vital capacity percent predicted (FVC%pred) [MD − 6.69, 95% confidence interval (CI) -7.73–-5.65, *P* < 0.001, *I*^*2*^ = 5%], and forced expiratory volume in 1 s/forced vital capacity (FEV_1_/FVC) (MD -3.76, 95% CI -4.58–-2.95,*P* < 0.001, *I*^*2*^ = 0%); in contrast, Charlson comorbidity index (MD 0.47, 95% CI 0.37–0.58, *P* < 0.001, *I*^*2*^ = 0], COPD assessment test (CAT) score (MD 5.61, 95% CI 4.62–6.60, *P* < 0.001, *I*^*2*^ = 80%), the quantity of cigarettes smoked (pack-years) (MD 3.09, 95% CI 1.60–4.58, *P* < 0.001, *I*^*2*^ = 41%), exacerbations in previous year (2.65, 95% CI 2.32–2.97, *P* < 0.001, *I*^*2*^ = 91%), modified Medical British Research Council (mMRC) score (MD 0.72, 95% CI 0.63–0.82, *P* < 0.001, *I*^*2*^ = 57%), and body mass index (BMI), obstruction, dyspnea, exacerbations (BODEx) (MD 1.78, 95% CI 1.28–2.28, *P* < 0.001, *I*^*2*^ = 91%), *I*^*2*^ = 34%) were significantly higher in patients with FE-CB phenotype. No significant between-group difference was observed with respect to BMI (MD-0.14, 95% CI -0.70–0.42, *P* = 0.62, *I*^*2*^ = 75%).

**Conclusion:**

COPD patients with the FE-CB phenotype had worse pulmonary function and higher CAT score, mMRC scores, frequency of acute exacerbations, and the quantity of cigarettes smoked (pack-years) than those with the NE phenotype.

Chronic obstructive pulmonary disease (COPD) is characterized as a heterogeneous disease [1–3]. The Spanish Guidelines for Management of Chronic Obstructive Pulmonary Disease (GesEPOC) attempt to identify and elaborate this heterogeneity by characterizing various phenotypes in order to guide individualized diagnosis and treatment. Since its publication in 2013, the guidelines have been gradually referred to by researchers in other countries and have been constantly updated. On the basis of the risk stratification and clinical manifestations, the GesEPOC 2017 have incorporated some modifications to the COPD phenotypes to better reflect the differences of various COPD phenotypes observed in clinical practice.

GesEPOC identifies four phenotypes: non-exacerbator (NE), asthma-COPD overlap (ACO), exacerbator with emphysema (FE-E), and frequent exacerbator with chronic bronchitis (FE-CB) [4, 5].

In our previous studies, we had explored the characteristics of the FE-CB phenotype and the ACO phenotype in COPD patients. However, the characteristics of the FE-CB phenotype and the NE phenotype in patients with COPD is still controversial [5].

The GesEPOC 2017 provides guidance for the diagnosis and treatment of patients with the FE-CB and NE phenotypes. Whether high-risk FE-CB patients or high-risk NE patients, initial treatment can choose the combination of long-acting β2-agonists and long-acting muscarinic antagonists. However, for high-risk patients with the FE-CB phenotype, the best treatment is guided by the individual characteristics of the patient. The optional drugs include inhaled corticosteroids, phlegm-resolving drugs, and antibiotics [4]. GesEPOC 2017 recommended long-term use of macrolide antibiotics to reduce the number of acute exacerbations in high-risk COPD patients who experienced more than three acute exacerbations in the past year [4].

However, these two phenotypes are not well characterized with respect to the epidemiology, risk factors, pathogenesis, clinical features, and prognosis. In terms of clinical characteristics, there is conflicting evidence of the association of these phenotypes with smoking, pulmonary function, COPD Assessment Test (CAT) score, frequency of acute exacerbations, body mass index (BMI), St. George’s questionnaire score (SGRQ), and complications. In a study, patients with FE-CB phenotype showed worse pulmonary function, higher CAT score, worse endurance to physical labor [6], and higher incidence of heart failure, anxiety, depression, and other complications [7]. Among all phenotypes, FE-CB was associated with more than three complications [8]. In some studies, patients with the FE-CB phenotype showed lower forced vital capacity percent predicted (FVC%pred) [9–14], forced expiratory volume in one second percent predicted (FEV_1_%pred) [9–17], forced expiratory volume in one second/ forced vital capacity (FEV_1_/FVC) [10–12, 14], and forced expiratory volume in 1 second (FEV_1_) [14, 17] as compared to those with the NE phenotype; however, other studies have revealed opposite results (FEV_1_%pred [18], FEV_1_/FVC [9], FEV_1_ [18]).

In this study, we sought to investigate the differences in smoking, pulmonary function, CAT, and BMI between COPD patients with the FE-CB phenotype and those with the NE phenotype; the objective was to better characterize the clinical features of these two phenotypes.

## Research methods

This meta-analysis was performed according to the Meta-Analysis of Observational Studies in Epidemiology (MOOSE) guidelines. Document retrieval and screening programs were established in advance.

### Search strategy

We searched CNKI, Wan fang, Chongqing VIP, China Biology Medicine disc, PubMed, Cochrane Library, and EMBASE databases from the times of their inception to April 30, 2019. The language was restricted to English or Chinese. Referring to our previous research [5], the research was obtained using the following keywords or combinations: “Chronic Obstructive Pulmonary Disease” or “COPD”; merging “Non-exacerbators” or “Nonexacerbators” or “nonexacerbator” or “non-frequent exacerbators with chronic bronchitis or emphysema” or “non-exacerbator phenotype with either chronic bronchitis or emphysema” or “NE” or “NONEX” or “NE-CB/E” or “NON-AE”, merging “frequent exacerbator(s) with chronic bronchitis” or “exacerbator(s) with chronic bronchitis” or “exacerbator phenotype with chronic bronchitis” or “FE-CB”. In order to avoid omissions, the references of relevant reviews and meta-analyses were manually screened.

### Inclusion and exclusion criteria

Inclusion criteria: 1) COPD patients; 2) the characteristics of FE-CB phenotype and NE phenotype were reported; 3) main outcomes: FEV_1_%pred, FEV_1_, FEV_1_/FVC, FVC%pred, FVC, and the diffusing capacity for carbon monoxide (DLCO). Secondary outcomes: smoking, body mass index (BMI), symptoms, frequency of acute exacerbations in previous year, CAT score, modified Medical Research Council (mMRC) dyspnea scale score, BMI, obstruction, dyspnea, exacerbations (BODEx) [or BMI, obstruction, dyspnea, exercise capacity (BODE)], complication, and Charlson comorbidity index. Studies were included only if they reported at least one of the main outcomes. 4) Cross-sectional observation study, case-control study, cohort study, clinical randomized trials, and semi-randomized trials.

Exclusion criteria: 1) repetitive articles; 2) plagiarized literature; 3) study design defects; 4) incomplete data or the inability to extract relevant data.

### Data extraction and quality assessment

The literature selection and data extraction were performe by two researchers (Jianjun Wu, Yingxue Zhang) independently. Disagreements were determined by discussion or by a third coauthor (Hong-ri Xu). The quality assessment was analyzed by Cross-Sectional/Prevalence Study Quality recommendations. The criterion contains 11 items. Each item was rated as “yes” (1 point), “no”(0 point), and “unclear” (0 point). The included studies were categorized as follows: low quality (0–3), moderate quality (4–7), and high quality (8–11).

### Observation indicators

The following information was extracted: the researchers (author name, date of publication, language, country, study type) and the research (sample size, average age, symptoms, pulmonary function, smoking, exacerbations in previous year, mMRC score, and other indexes).

### Publication bias assessment

When more than ten studies were included in the meta-analysis, we evaluated potential publication bias by funnel plots and quantified by the Begg [19] and the Harbord [20] tests.

### Data analysis

The statistical analyses were conducted using Rev. Man 5.3. Continuous variables were evaluated using the mean difference (MD) with 95% confidence intervals (CIs). Dichotomous variables were evaluated using the odds ratio (OR) or relative risk (RR) with 95% CIs. *P* < 0.05 was considered statistically significant. The heterogeneity was evaluated by *I*^*2*^. If the heterogeneity was not significant (*P* > 0.1 and *I*^2^ < 50%), the fixed effect model was used. If the heterogeneity was significant (*P* < 0.1 and *I*^2^ > 50%), the random-effects model was used.

### Literature search

**Fig. 1:**
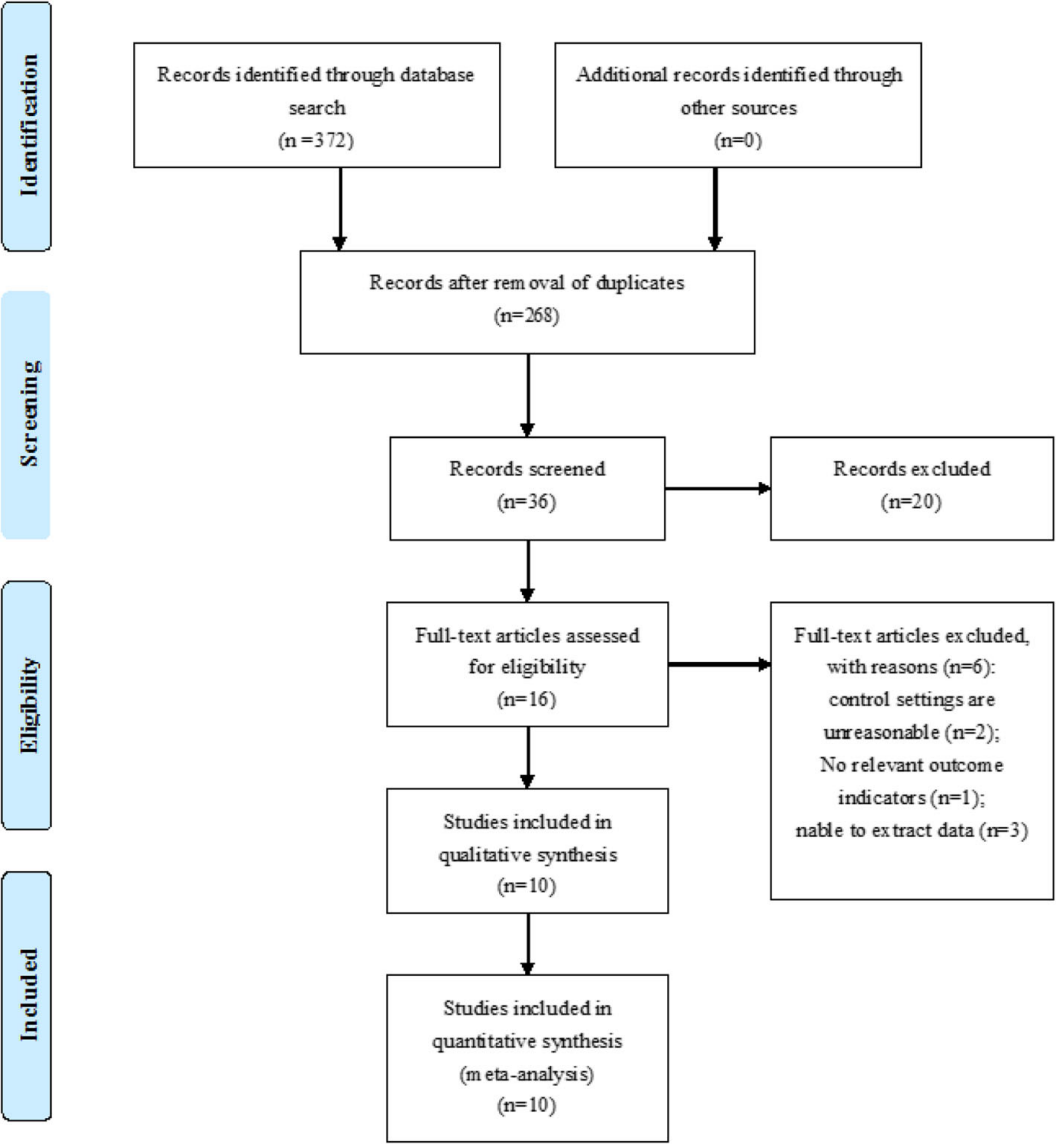
Schematic illustration of the study design and the study selection criteria

Three hundred seventy-two articles were retrieved initially through electronic database searching and manual search. Three hundred fifty-sixstudies were excluded after reading titles and abstracts. After a full-text review, 10 studies met the inclusion criteria and were included in the meta-analysis. The screening procedure is illustrated in Fig. 1, Additional file 1: Flow Diagram, Table S1, and Text S1.

### Basic characteristics of the included studies

Eight thousand eight hundred forty-nine patients from ten studies [9–18] were included, of which 2699 patients with the FE-CB phenotype and 6150 patients with the NE phenotype. Study characteristics are summarized in Table 1.

**Table 1:** Basic characteristics of the studies included in the meta-analysis

Author	Year	Country	Language	Research type	Cases (FE-CB/NE)	Gender (male) (FE-CB/NE)	Age (years) (FE-CB/NE)	Evaluation indices
Alcázar-Navarrete, B.	2016	Spain	English	Cross-sectional observation study	34/34	32/29	72 ± 10.471 ± 9.9	FEV_1_%, FEV_1_/FVC, FVC%, BMI, the quantity of cigarettes smoked (pack-years), CAT score
Arkhipov, V.	2017	Russia	English	Cross-sectional observation study	415/398	356/347	64.6 ± 8.564.7 ± 8.9	FEV_1_%, the quantity of cigarettes smoked (pack-years), BMI, CAT score
Calle Rubio, M.	2017	Spain	English	Cross-sectional observation study	188/307	157/255	69.5 ± 8.667.2 ± 9.3	FEV_1_, FEV_1_%, the quantity of cigarettes smoked (pack-years), CAT, mMRC, BODEx, exacerbations in previous year, BMI
Chee-Shee Chai	2019	Malaysia	English	Cross-sectional observation study	75/54	70/50	70.7 ± 9.274.1 ± 8.1	FEV_1_%, the quantity of cigarettes smoked (pack-years), CAT, mMRC, exacerbations in previous year
Corlateanu, A.	2017	Moldova	English	Cross-sectional observation study	138/175	–		FVC%, FEV_1_%, FEV_1_/FVC, CAT
Cosio, B. G.	2016	Spain	English	Cross-sectional observation study	99/550	85/460	69.5 ± 8.167.4 ± 9.1	FEV_1_%, FVC%, FEV_1_/FVC, the quantity of cigarettes smoked (pack-years), BMI, CAT, Charlson comorbidity index
Golpe, R.	2018	Spain	English	Cross-sectional observation study	194/531	167/433	72.7 ± 8.968.5 ± 9.5	FEV_1_%, FVC%, FEV_1_/FVC, BMI, BODEx, Charlson comorbidity index
Koblizek, V.	2017	Czech	English	Cross-sectional observation study	687/2125	494/1500	66.6 ± 8.366.3 ± 8.7	FEV_1_%, FVC%, BMI, CAT, mMRC, exacerbations in previous year
Miravitlles, M.	2015	Germany	English	Cross-sectional observation study	602/1894	514/1617	69.3 ± 9.266.6 ± 9.7	FEV_1_%, FVC%, FEV_1_, FVC, FEV_1_/FVC, mMRC, exacerbations in previous year, BODEx, CAT, BMI, the quantity of cigarettes smoked (pack-years), Charlson comorbidity index
Pan Qing	2016	China	Chinese	Cross-sectional observation study	267/82	217/68	76 ± 10.061 ± 6.4	FEV_1_、FEV_1_%

FE-CB: frequent exacerbators with chronic bronchitis; NE: non-exacerbator; FVC: forced vital capacity; FEV_1_: forced expiratory volume in one second; CAT: COPD assessment test; BMI: body mass index; mMRC: modified British Medical Research Council dyspnea scale;BODEx: BMI, obstruction, dyspnea, exacerbations; FVC%pred: forced vital capacity percent predicted; FEV_1_%pred: forced expiratory volume in one second percent predicted

### Quality evaluation

Of the 10 studies included, 7 were moderate quality and 3 were high quality. AS show in Table 2.

**Table 2:** Methodological quality evaluation of studies included

Study ID	1	2	3	4	5	6	7	8	9	10	11	Total
Alcázar-Navarrete, B.2016	+	+	–	–	+	+	–	+	–	–	–	5
Arkhipov, V.2017	+	+	+	–	+	+	+	+	–	+	–	8
Calle Rubio, M.2017	+	+	+	–	+	+	–	+	–	–	–	6
Chee-Shee Chai2019	+	+	+	–	+	+	+	–	–	–	–	6
Corlateanu, A.2017	+	+	–	–	+	+	–	+	–	–	–	5
Cosio, B. G.2016	+	+	+	–	+	+	+	+	–	–	+	8
Golpe, R.2018	+	+	+	–	+	+	+	+	–	–	+	8
Koblizek, V.2017	+	+	+	–	+	+	+	+	–	–	–	7
Miravitlles, M.2015	+	+	–	–	+	+	+	+	–	–	–	6
Pan Qing2016	+	+	+	–	+	+	–	+	–	–	–	6

Note: +:YES; −:NO; 0:not clear. 1. Define the source of information (survey, record review); 2. List inclusion and exclusion criteria for exposed and unexposed subjects (cases and controls) or refer to previous publications; 3. Indicate time period used for identifying patients; 4. Indicate whether subjects were consecutive, if not population-based; 5. Indicate if evaluators of subjective components of study were masked to other aspects of the status of the participants; 6. Describe any assessments undertaken for quality assurance purposes (e.g., test/retest of primary outcome measurements); 7. Explain any patient exclusions from analysis; 8. Describe how confounding was assessed and/or controlled; 9. If applicable, explain how missing data were handled in the analysis; 10. Summarize patient response rates and completeness of data collection; 11. Clarify what follow-up, if any, was expected and the percentage of patients for which incomplete data or follow-up was obtained

### Comparison of the characteristics of COPD patients between the FE-CB and the NE phenotypes

#### FEV_1_%pred

**Fig. 2:**
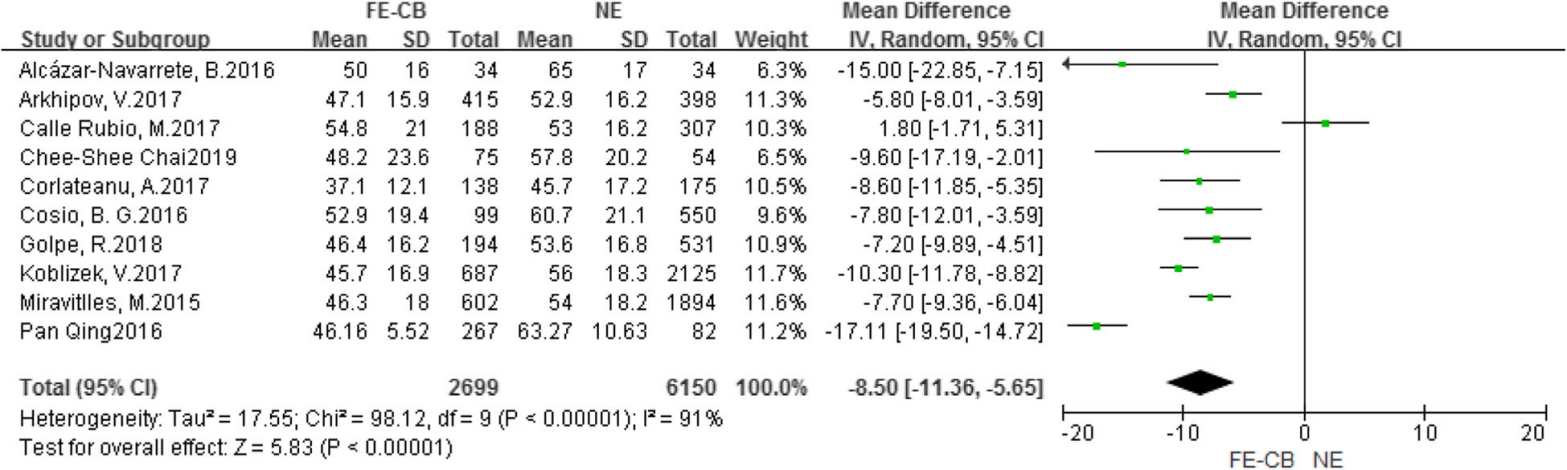
Difference of FEV_1_%pred between the FE-CB and the NE phenotypes

As shown in Fig. 2, ten included studies [9–18] had reported FEV_1_%pred. Nine studies [9–17] had reported that compared with the NE phenotype, the FEV_1_%pred of the FE-CB phenotype was lower. However, one other study [18] showed that there was no significant between the two phenotype. Meta-analysis showed that compared with the NE phenotype, the FEV_1_%pred of the FE-CB phenotype was lower (MD -8.50, 95% CI -11.36–-5.65, *P* < 0.001, *I*^*2*^ = 91%) (Fig. 2). Sensitivity analysis revealed that the heterogeneity was belonged to the studies by Calle Rubio et al. [18], Qing et al. [17], and Koblizek et al. [13] After excluding these studies, the result showed that compared with the NE phenotype, the FEV_1_%pred of the FE-CB phenotype was lower (MD -7.46, 95% CI -8.52–-6.40, *P* < 0.001, *I*^*2*^ = 10%) (Additional file 1: Figure S1).

#### FEV_1_

Three included studies [14, 17, 18] had reported FEV_1_. The heterogeneity among the samples was large, and only descriptive analysis was done. In two studies [14, 17], the FEV_1_ of the FE-CB phenotype was significantly lower than that of the NE phenotype, while one other study [18] found no significant between-group difference in this respect.

#### FVC%pred

**Fig. 3:**
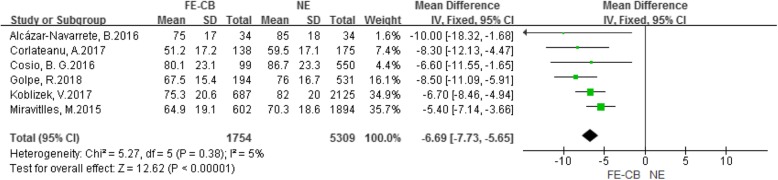
Difference of FVC%pred between the FE-CB and the NE phenotypes

As shown in Fig. 3, six included studies [9–14] had reported the FVC%pred. All six studies had reported that compared with the NE phenotype, the FVC% of FE-CB phenotype was lower. There was no significant heterogeneity among the studies. The fixed-effect model was used for analysis. Meta-analysis showed that compared with the NE phenotype, the FVC%pred of COPD patients with the FE-CB phenotype was significantly lower (MD -6.69, 95% CI -7.73–-5.65, *P* < 0.001, *I*^*2*^ = 5%) (Fig. 3).

#### FEV_1_/FVC

**Fig. 4:**
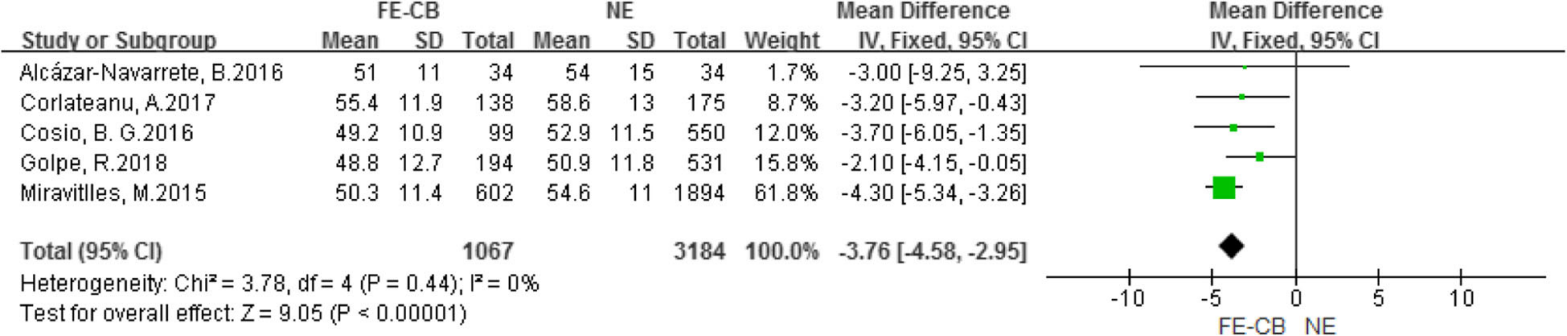
Difference of FEV_1_/FVC between the FE-CB and the NE phenotypes

As shown in Fig. 4, five included studies [9–12, 14] had reported FEV_1_/FVC. Three studies [11, 12, 14] had reported that compared with the NE phenotype, the FEV_1_/FVC of the FE-CB phenotype was lower. However, other two studies [9, 10] showed that there was no significant between the two phenotype. Meta-analysis showed that compared with the NE phenotype, the FEV_1_/FVC of FE-CB phenotype was lower (MD -3.76, 95% CI -4.58–-2.95, *P* < 0.001, *I*^*2*^ = 0%) (Fig. 4).

#### The quantity of cigarettes smoked (pack-years), exacerbations in previous year, CAT score, BMI, BODEx, Charlson comorbidity index, mMRC score

All details of outcomes could be found in Table 3, Additional file 1: Figures S2–10, Table S2.

**Table 3:** Difference of other indexes between the NE and FE-CB phenotypes

Secondary outcomes	included studies	MD	95% CI	***P***	***I***^***2***^, ***P***
the quantity of cigarettes smoked (pack-years)	Six studies	3.09	(1.60,4.58)	< 0.001	41%, 0.14
CAT score	Eight studies	5.61	(4.62,6.60)	< 0.001	80%, < 0.001
mMRC score	Four studies	0.72	(0.63,0.82)	< 0.001	57%, 0.07
BMI	Seven studies	-0.14	(−0.70,0.42)	0.62	75%, < 0.001
Charlson comorbidity index	Three studies	0.47	(0.37,0.58)	< 0.001	0, 0.70
BODEx	Three studies	1.78	(1.28,2.28)	< 0.001	91%, < 0.001

CI: confidence interval; CAT: COPD assessment test; BMI: body mass index; mMRC: modified British Medical Research Council dyspnea scale; BODEx: BMI, obstruction, dyspnea, exacerbations

## Discussion

In this study, COPD patients with the FE-CB phenotype had worse FEV_1_%, FEV_1_/FVC, and FVC% than those with the NE phenotype. In addition, patients with the FE-CB phenotype had significantly higher CAT score, the quantity of cigarettes smoked (pack-years), number of acute exacerbations, and mMRC score.

Pulmonary function tests play an important role in the diagnosis and treatment of COPD. Airway obstruction assessed by spirometry should follow the reference values provided by the European Respiratory Society (ERS) Global Lung Initiative (GLI) [21]. In addition, pulmonary function tests should include the assessment of pulmonary hyperinflation and emphysema using whole body plethysmography and the determination of diffusion capacity. This is important because both lung hyperinflation and emphysema can occur without overt airway obstruction [21]. In clinical settings, pulmonary function tests are also widely used to evaluate the degree of airflow limitation, to monitor disease progression, and to evaluate the therapeutic response. However, the diagnostic and prognostic relevance of pulmonary function tests in the context of COPD has been constantly questioned. At present, we use FVE_1_/FVC < 70% after inhalation of bronchodilator as the gold standard for diagnosis of obstructive ventilation function. However, due to considerable variability in pulmonary function itself, many authors have proposed that the lowest limit of normal and the highest limit of normal should be considered as the lowest and the highest threshold, respectively. Theoretically, these are the most scientific evaluation criteria and have been endorsed by the American Thoracic Society (ATS)/European Respiratory Society (ERS) and the American Medical Association [22]. However, a study found that basic pulmonary function of COPD patients was not related to the therapeutic response to lung rehabilitation. The degree of baseline pulmonary function was not found to predict individual improvement in dyspnea, motor performance, activities of daily living, emotional state, or disease-specific health status after lung rehabilitation. These findings suggest that baseline pulmonary function cannot be used to identify good responders to lung rehabilitation therapy; therefore, the results of pulmonary function tests cannot be used as a criterion to recommend lung rehabilitation for COPD patients [23]. Thus, pulmonary function is not enough to capture the heterogeneity of COPD, and there are some limitations of its use to guide individual diagnosis and treatment [24]. At present, the pulmonary function characteristics of COPD patients with different phenotypes are still unclear. This study found that the pulmonary function of patients with the FE-CB phenotype is worse than that of NE phenotype, mainly with respect to FEV_1_%pred, FVE_1_/FVC, and FVC%pred. This may be attributable to the higher frequency of acute exacerbations in patients with the FE-CB phenotype, which results in a decline in pulmonary function. However, no positive results were found with respect to FEV_1,_ which may be related to the small sample size or to the large variability of FEV_1_ per se. In addition, the analysis of FEV_1_%pred was affected by considerable heterogeneity, which may be related to the large variability of FEV_1_ per se as well as the selected samples.

Cigarette smoke exposure is one of the major risk factors for COPD [25]. However, a considerable proportion of non-smokers (25–45%) develop COPD [26]. In addition, exposure to both maternal and own smoking was associated with lower FEV_1_/FVC and higher risk of hospitalization/death from COPD than their independent associations [26]. The association between maternal smoking and COPD is influenced by the duration of smoking exposure. However, among non-smokers, there is no strong evidence that maternal smoking affects adult lung health [26]. In this study, six studies [9, 11, 14–16, 18] had reported the quantity of cigarettes smoked (pack-years). One study [14] found that the number of the quantity of cigarettes smoked (pack-years) of the FE-CB phenotype was higher than that of the NE phenotype (*P* < 0.05), while five studies [9, 11, 15, 16, 18] found no significant between them. Meta-analysis showed that the quantity of cigarettes smoked (pack-years) of the FE-CB phenotype was significantly higher than that of the NE phenotype (MD 3.09, 95% CI 1.60–4.58, *P* < 0.001, *I*^*2*^ = 41%) (Additional file 1: Figure S2).

The Global Initiative for Chronic Obstructive Lung Disease (GOLD) 2017 guidelines recommend the use of CAT or mMRC scale scores to assess symptoms in COPD patients [24]. The CAT questionnaire was used to assess and quantify the health-related quality of life and symptom burden of COPD patients. It consists of 8 questions with a total score of 40 points. In the mMRC dyspnea scale, the severity of dyspnea is rated on a 5-point scale (0–4). The GOLD guidelines recommend the use of CAT score 10 or MMRC score 2 as the threshold level for symptoms [24]. However, some studies have shown discrepancy between the CT and MRC scales for assessment of severity of COPD. The main reason may be that CAT also includes many aspects of quality of life, while mMRC only reflects the degree of dyspnea and does not take cognizance of other important symptoms of COPD, such as cough, sputum, chest tightness, and depression [27]. In another study, compared with other COPD phenotypes, the patients of FE-CB phenotype suffered lower exercise endurance and higher CAT score, while the patients of NE phenotype owned lower CAT score, higher lung function, and fewer symptoms [7]. The conclusion is similar to that of the present study. In this study, eight studies [9–11, 13–16, 18] reported CAT scores. In all eight studies, the CAT score of COPD patients with the FE-CB phenotype was significantly higher than that of the NE phenotype. Meta-analysis (random-effects model) showed that CAT score of patients with the FE-CB phenotype was higher than that of patients with the NE phenotype (MD 5.61, 95% CI 4.62–6.60, *P* < 0.001, *I*^*2*^ = 80%) (Additional file 1: Figure S3). Sensitivity analysis revealed that the heterogeneity was belonged to the studies by Calle Rubio et al. [18] and Corlateanu et al. [10] After excluding these studies, CAT score of COPD patients with the FE-CB phenotype was significantly higher than that of patients with the NE phenotype (MD 5.73, 95% CI 5.32–6.14, *P* < 0.001, *I*^*2*^ = 38%)(Additional file 1: Figure S4). Four studies [13, 14, 16, 18] reported mMRC scores. In all 4 studies, the mMRC score of COPD patients with the FE-CB phenotype was significantly higher than that of the NE phenotype. Meta-analysis showed that compared with the NE phenotype, the mMRC score of the FE-CB phenotype was higher (MD 0.72, 95% CI 0.63–0.82, *P* < 0.001, *I*^*2*^ = 57%) (Additional file 1: Figure S5). Sensitivity analysis revealed that the heterogeneity was belonged to the study by Miravitlles et al. [14] After excluding these study, the mMRC score of the FE-CB phenotype was still higher than that of the NE phenotype (MD 0.68, 95% CI 0.61–0.75, *P* < 0.001, *I*^*2*^ = 17%) (Additional file 1: Figure S6). We observed a consistency between the CAT and mMRC scores for evaluating the symptoms of patients with different phenotypes of COPD.

Compared with individuals with higher BMI, those with lower BMI are more likely to suffer from COPD and have lower lung function [28]. Previous studies had explored the characteristic of BMI in COPD patients with the emphysema phenotype and the bronchitis phenotype. Compared with the chronic bronchitis phenotype, patients with the emphysema phenotype had lower BMI [5]. However, it is not clear whether there is a difference in BMI between FE-CB and NE phenotypes of COPD patients. In this study, seven studies reported BMI. In one study [13], BMI was lower in COPD patients with the NE phenotype than in COPD patients with the FE-CB phenotype. One other study [18] reported the opposite relationship, while the remaining five studies [9, 11, 12, 14, 15] showed that there was no difference between the two phenotypes. Meta-analysis showed that BMI of COPD patients with the NE phenotype was not different from that of the FE-CB phenotype (MD -0.14, 95% CI -0.70–0.42, *P* = 0.62, *I*^*2*^ = 75%) (Additional file 1: Figure S7). Sensitivity analysis indicated that the heterogeneity was mainly attributable to the studies by Calle Rubio et al. [18] and Koblizek et al. [13]. After excluding these studies, there was no significant between-group difference with respect to BMI (MD -0.05, 95% CI -0.36–0.26, *P* = 0.77, *I*^*2*^ = 24%)(Additional file 1: Figure S8).

Four included studies [13, 14, 16, 18] had reported the exacerbations in previous year. The heterogeneity among the samples was large, and only descriptive analysis was done. In all four studies, the exacerbations in previous year of the FE-CB phenotype was significantly higher than that of the NE phenotype.

This study also found that compared with the NE phenotype patients, BODEx (Additional file 1: Figure S9), and Charlson comorbidity index (Additional file 1: Figure S10) of FE-CB phenotype patients were higher; however, due to few sample size, further research is required to draw more definitive conclusions.

### Strengths of this study

COPD is character as a heterogeneous disease [29–32]. Phenotype is helpful to recognize the heterogeneity and understand the evolution of disease [30, 32]. Phenotype helps guide diagnosis and treatment [30, 32]. In this study, the characteristics of patients with FE-CB and NE were studied by meta-analysis, which would help to more comprehensively describe the characteristics of FE-CB and NE of COPD and provide basis for diagnosis and treatment of COPD. This study was helpful to provide early warning and guidance for patients with FE-CB and NE phenotypes. For example, patients with poor lung function might have frequent acute exacerbations. The patients with FE-CB phenotype might be accompanied by poor lung function, and such patients might be more likely to benefit from lung rehabilitation exercise.

### Limitations of this study

In this study, we compared the FEV_1_%, FVC%, FEV_1_/FVC, FEV_1_, CAT score, BMI, mMRC score, the quantity of cigarettes smoked (pack-years), and the number of acute exacerbations between COPD patients with the FE-CB phenotype and the NE phenotype. However, we did not discuss the differences in race, gender, age, symptoms and complications between the two COPD phenotypes. In addition, we did not do stratified studies on these two phenotypes, such as studies on different GOLD comprehensive assessment grades (A, B, C, D). These elements need to be studied in a future study.

The survey included eight studies in Europe and two in Asia. The absence of studies that met the inclusion criteria in Africa, America and Oceania is another limitation of our analysis.

In addition, some variables in this study changed with time. For example, lung function changed with the development of the disease [33]. The change of lung function might be accompanied by a series of other characteristics, such as the aggravation of wheezing symptoms, and then the increase of CAT score and MMRC score. For patients with NE phenotype, this might be a warning. If the patient’s lung function continued to decline, accompanied by the increase of CAT score and MMRC score, then the patient might become a patient with FE-CB phenotype. The treatment focus and prognosis of this patient might be different. But for patients in the FE-CB phenotype, the warning effect might be smaller. If the patient’s lung function continued to decline, it might be accompanied by an increase in CAT score, MMRC score, and the number of acute exacerbations. However, the patient was always in the group with FE-CB phenotype. The treatment focus and prognosis of this patient might not change. In this study, those indicators with dynamic changes had not been discussed. These elements need to be studied in a future study.

## Conclusion

Compared with COPD patients with the NE phenotype, COPD patients with the FE-CB phenotype had worse lung function, higher CAT score, the quantity of cigarettes smoked (pack-years), frequency of acute exacerbation, and mMRC scores.

## Supplementary information

**Additional file 1: Figure S1.** Sensitivity analysis of Difference of FEV_1_%pred between the FE-CB and the NE phenotypes. Forest plots of the sensitivity analysis for difference of FEV_1_%pred between the FE-CB and the NE phenotypes. **Figure S2.** Difference of the quantity of cigarettes smoked (pack-years) between the FE-CB and the NE phenotypes. Forest plots of the difference of the quantity of cigarettes smoked (pack-years) between the FE-CB and the NE phenotypes. **Figure S3.** Difference of CAT score between the FE-CB and the NE phenotypes. Forest plots of the difference of cat score between the FE-CB and the NE phenotypes. **Figure S4.** Sensitivity analysis of CAT between the FE-CB and the NE phenotypes. Forest plots of the sensitivity analysis for CAT between the FE-CB and the NE phenotypes. Difference of mMRC score between the FE-CB and the NE phenotypes. Forest plots of the difference of mMRC score between the FE-CB and the NE phenotypes. **Figure S6.** Sensitivity analysis of mMRC between the FE-CB and the NE phenotypes. Forest plots of the sensitivity analysis for mMRC between the FE-CB and the NE phenotypes. **Figure S7.** Difference of BMI between the FE-CB and the NE phenotypes. Forest plots of the difference of BMI between the FE-CB and the NE phenotypes. **Figure S8.** Sensitivity analysis of BMI between the FE-CB and the NE phenotypes. Forest plots of the sensitivity analysis of BMI between the FE-CB and the NE phenotypes. **Figure S9.** Difference of BODEx between the FE-CB and the NE phenotypes. Forest plots of the difference of BODEx between the FE-CB and the NE phenotypes. **Figure S10.** Difference of Charlson comorbidity index between the FE-CB and the NE phenotypes. Forest plots of the difference of Charlson comorbidity index between the FE-CB and the NE phenotypes. **Flow Diagram.** PRISMA 2009 Flow Diagram. The screening procedure the study. **Table S1.** Excluded list. List of excluded full-text articles. **Table S2.** Other indices. Other indices in different phenotyp. **Text S1.** Literature Search. The full details of the databases searched to identify the studies.

## Data Availability

All data will be available by personal communication with corresponding author.

## Abbreviations

COPD: chronic obstructive pulmonary disease
FVC%pred: forced vital capacity percent predicted
FEV_1_%pred: forced expiratory volume in one second percent predicted
FEV_1_: forced expiratory volume in one second
FVC: forced vital capacity
FEV_1_/FVC: forced expiratory volume in one second/forced vital capacity
FE-C: frequent exacerbator with chronic bronchitis phenotype
NE: non-exacerbator phenotype
BMI: body mass index
CAT: COPD assessment test score
mMRC: modified Medical British Research Council
BODEx: BMI, obstruction, dyspnea, exacerbations
MD: mean
CI: confidence interval

## References

[1] Sidhaye VK, Nishida K, Martinez FJ (2018). Precision medicine in COPD: where are we and where do we need to go?. Eur Respir Rev.

[2] Di Stefano A, Coccini T, Roda E (2018). Blood MCP-1 levels are increased in chronic obstructive pulmonary disease patients with prevalent emphysema. Int J Chronic Obstructive Pulmonary Disease.

[3] Martinez FJ, Han MK, Allinson JP (2018). At the root: defining and halting progression of early chronic obstructive pulmonary disease. Am J Respir Crit Care Med.

[4] Miravitlles M, Soler-Cataluña JJ, Calle M (2017). Spanish guidelines for Management of Chronic Obstructive Pulmonary Disease (GesEPOC)2017.Pharmacological treatment of stable phase. Arch Bronconeumol.

[5] Wu J-J, Xu H-R, Zhang Y-X (2019). The characteristics of the frequent Exacerbators with chronic bronchitis phenotype and the asthma-chronic obstructive pulmonary disease overlap syndrome phenotype in chronic obstructive pulmonary disease patients: a meta-analysis and system review. Medicine (Baltimore).

[6] Kania A, Krenke R, Kuziemski K (2018). Distribution and characteristics of COPD phenotypes-results from the polish sub-cohort of the POPE study. Int J Chronic Obstructive Pulmonary Disease.

[7] Reiger G, Zwick R, Lamprecht B (2018). Phenotypes of COPD in an Austrian population: national data from the POPE study. Wien Klin Wochenschr.

[8] Bernardino A-N, Antonio TJ, Antonio RJ (2018). Geographic variations of the prevalence and distribution of COPD phenotypes in Spain: “the ESPIRAL-ES study”. Oxidative Med Cell Longev.

[9] Alcázar-Navarrete B, Romero-Palacios PJ, Ruiz-Sancho A (2016). Diagnostic performance of the measurement of nitric oxide in exhaled air in the diagnosis of COPD phenotypes. Nitric Oxide - Biology and Chemistry.

[10] Corlateanu A, Botnaru V, Rusu D (2017). Assessment of health-related quality of life in different phenotypes of COPD. Current Respiratory Medicine Reviews.

[11] Cosio BG, Soriano JB, López-Campos JL (2016). Correction: distribution and outcomes of a phenotype-based approach to guide COPD management: results from the CHAIN cohort. PLoS One.

[12] Golpe R, Suárez-Valor M, Martín-Robles I (2018). Mortality in COPD patients according to clinical phenotypes. Int J Chronic Obstructive Pulmonary Disease.

[13] Koblizek V, Milenkovic B, Barczyk A (2017). Phenotypes of COPD patients with a smoking history in central and Eastern Europe: the POPE study. Eur Respir J.

[14] Miravitlles M, Barrecheguren M, Román-Rodríguez M (2015). Frequency and characteristics of different clinical phenotypes of chronic obstructive pulmonary disease. Int J Tuberculosis Lung Disease.

[15] Arkhipov V, Arkhipova D, Miravitlles M (2017). Characteristics of COPD patients according to GOLD classification and clinical phenotypes in the Russian Federation: the SUPPORT trial. Int J Chronic Obstructive Pulmonary Disease.

[16] Chai C-S, Liam C-K, Pang Y-K (2019). Clinical phenotypes of COPD and health-related quality of life: a cross-sectional study. Int J COPD.

[17] Pan Qing,Lv Zhifang.clinical application value of the new guide patients with chronic obstructive pulmonary disease. J Shandong University (Health Sciences), 2016, 54(3): 63–7.

[18] Calle Rubio M, Casamor R, Miravitlles M (2017). Identification and distribution of COPD phenotypes in clinical practice according to Spanish COPD guidelines: the FENEPOC study. In tJ Chronic Obstructive Pulmonary Disease.

[19] Begg CB, Mazumdar M (1994). Operating characteristics of a rank correlation test for publication bias. Biometrics.

[20] Harbord RM, Egger M, Sterne JA (2006). A modified test for small-study effects in meta-analyses of controlled trials with binary endpoints. Stat Med.

[21] Windisch W (2018). Criée CP.COPD-importance of lung function testing for diagnosis and treatment. Dtsch Med Wochenschr.

[22] Lei Zhu,Lijuan Hu,Li li,et al. Suggestions on diagnosis of pulmonary function. Chin J Tubere Respir Dis, 2018, 41(4): 308–311.

[23] Augustin IML,Wouters EFM,Houben-Wilke S,et al. Comprehensive Lung Function Assessment Does not Allow to Infer Response to Pulmonary Rehabilitation in Patients with COPD. J Clin Med, 2019, 8(1): pii: E27. 10.3390/jcm8010027 PMC6352188 30591662

[24] GOLD. Global Strategy for the Diagnosis, Management and Prevention of COPD, Global Initiative for Chronic Obstructive Lung Disease (GOLD) 2018. ; 2018 (https://goldcoped.org/. Accessed 17 July 2018).

[25] Hatipoğlu U (2018). Chronic obstructive pulmonary disease: more than meets the eye. Ann Thoracic Med.

[26] Magnus MC, Henderson J, Tilling K (2018). Independent and combined associations of maternal and own smoking with adult lung function and COPD. Int J Epidemiol.

[27] Cheng SL, Lin CH, Wang CC (2019). Comparison between COPD assessment test (CAT) and modified Medical Research Council (mMRC) dyspnea scores for evaluation of clinical symptoms, comorbidities and medical resources utilization in COPD patients. J Formos Med Assoc.

[28] Grigsby MR, Siddharthan T, Pollard SL (2019). Low Body Mass Index Is Associated with Higher Odds of COPD and Lower Lung Function in Low- and Middle-Income Countries. Copd.

[29] Mansoor S, Obaida Z, Ballowe L (2020). Clinical impact of multidisciplinary outpatient care on outcomes of patients with COPD. Int J Chron Obstruct Pulmon Dis.

[30] Guerreiro I, Soccal PM (2019). COPD and phenotypes. Rev Med Suisse.

[31] Pikoula M, Quint JK, Nissen F (2019). Identifying clinically important COPD sub-types using data-driven approaches in primary care population based electronic health records. BMC Med Inform Decis Mak.

[32] Radovanovic D, Contoli M, Marco FD (2019). Clinical and functional characteristics of COPD patients across GOLD classifications: results of a multicenter observational study. COPD.

[33] Jo YS, Kim SK, Park SJ (2019). Longitudinal change of FEV1 and inspiratory capacity: clinical implication and relevance to exacerbation risk in patients with COPD. Int J Chron Obstruct Pulmon Dis.

