# Supplementary material for: The characteristics of the frequent exacerbator with chronic bronchitis phenotype and non-exacerbator phenotype in patients with chronic obstructive pulmonary disease: a meta-analysis and system review
Source: BMC Pulm Med. 2020 Apr 23;20:103. doi: 10.1186/s12890-020-1126-x (PMC7181594; doi:10.1186/s12890-020-1126-x)
Supplement: Supplementary file 1 — Additional file 1: Figure S1. Sensitivity analysis of Difference of FEV1%pred between the FE-CB and the NE phenotypes. Forest plots of the sensitivity analysis for difference of FEV1%pred between the FE-CB and the NE phenotypes. Figure S2. Difference of the quantity of cigarettes smoked (pack-years) between the FE-CB and the NE phenotypes. Forest plots of the difference of the quantity of cigarettes smoked (pack-years) between the FE-CB and the NE phenotypes. Figure S3. Difference of CAT score between the FE-CB and the NE phenotypes. Forest plots of the difference of cat score between the FE-CB and the NE phenotypes. Figure S4. Sensitivity analysis of CAT between the FE-CB and the NE phenotypes. Forest plots of the sensitivity analysis for CAT between the FE-CB and the NE phenotypes. Difference of mMRC score between the FE-CB and the NE phenotypes. Forest plots of the difference of mMRC score between the FE-CB and the NE phenotypes. Figure S6. Sensitivity analysis of mMRC between the FE-CB and the NE phenotypes. Forest plots of the sensitivity analysis for mMRC between the FE-CB and the NE phenotypes. Figure S7. Difference of BMI between the FE-CB and the NE phenotypes. Forest plots of the difference of BMI between the FE-CB and the NE phenotypes. Figure S8. Sensitivity analysis of BMI between the FE-CB and the NE phenotypes. Forest plots of the sensitivity analysis of BMI between the FE-CB and the NE phenotypes. Figure S9. Difference of BODEx between the FE-CB and the NE phenotypes. Forest plots of the difference of BODEx between the FE-CB and the NE phenotypes. Figure S10. Difference of Charlson comorbidity index between the FE-CB and the NE phenotypes. Forest plots of the difference of Charlson comorbidity index between the FE-CB and the NE phenotypes. Flow Diagram. PRISMA 2009 Flow Diagram. The screening procedure the study. Table S1. Excluded list. List of excluded full-text articles. Table S2. Other indices. Other indices in different phenotyp. Text S1. Litera [file 12890_2020_1126_MOESM1_ESM.zip › Text S1R4.docx]

**Literature Search**

**The full details of the databases searched to identify the studies.**

The CNKI, Wanfang, Chongqing VIP, China Biology Medicine disc, PubMed, Cochrane Library and EMBASE were explored using broad search strategies to identify all study evaluating the difference of pulmonary function indices between chronic obstructive pulmonary disease (COPD) patients with the exacerbator with chronic bronchitis (FE-CB) phenotype and those with the asthma-COPD overlap syndrome (ACO) phenotype. All searches were run from the earliest date available (1966 for PUBMED, 1970 for Web of Science, 1988 for EMBASE) until April 30, 2019. All indexed journals were included and retrieved. In addition, Google Scholar was also used to search relevant study. In order to search and include all potential studies, we applied various combinations of the following medical subject headings and key words in order to hold high sensitivity:

**Search Items:** “Chronic Obstructive Pulmonary Disease” or “COPD”; merging “Non-exacerbators” or “Nonexacerbators” or “nonexacerbator” or “non-frequent exacerbators with chronic bronchitis or emphysema” or “non-exacerbator phenotype with either chronic bronchitis or emphysema” or “NE” or “NONEX” or “NE-CB/E” or “NON-AE”, merging “frequent exacerbator(s) with chronic bronchitis” or “exacerbator(s) with chronic bronchitis” or “exacerbator phenotype with chronic bronchitis” or “FE-CB”.

All the general medical journals subscribed by our university has been searched. We therefore hand searched BMJ, AMA, Elsevier, Science Online, Springer and Nature from library. We routinely reviewed the content of those medical journals for the year up until the end of April 30, 2019.Chinese papers were selected by searching WanFang Data, Chongqing VIP (CQVIP), China National Knowledge Infrastructure(CNKI) databases using the same search terms. The references of the eligible articles were also inspected to find other potential studies. As a search limit, only studies published in English or Chinese were included.
