# Supplementary material for: The characteristics of the frequent exacerbator with chronic bronchitis phenotype and non-exacerbator phenotype in patients with chronic obstructive pulmonary disease: a meta-analysis and system review
Source: BMC Pulm Med. 2020 Apr 23;20:103. doi: 10.1186/s12890-020-1126-x (PMC7181594; doi:10.1186/s12890-020-1126-x)
Supplement: Supplementary file 1 — Additional file 1: Figure S1. Sensitivity analysis of Difference of FEV1%pred between the FE-CB and the NE phenotypes. Forest plots of the sensitivity analysis for difference of FEV1%pred between the FE-CB and the NE phenotypes. Figure S2. Difference of the quantity of cigarettes smoked (pack-years) between the FE-CB and the NE phenotypes. Forest plots of the difference of the quantity of cigarettes smoked (pack-years) between the FE-CB and the NE phenotypes. Figure S3. Difference of CAT score between the FE-CB and the NE phenotypes. Forest plots of the difference of cat score between the FE-CB and the NE phenotypes. Figure S4. Sensitivity analysis of CAT between the FE-CB and the NE phenotypes. Forest plots of the sensitivity analysis for CAT between the FE-CB and the NE phenotypes. Difference of mMRC score between the FE-CB and the NE phenotypes. Forest plots of the difference of mMRC score between the FE-CB and the NE phenotypes. Figure S6. Sensitivity analysis of mMRC between the FE-CB and the NE phenotypes. Forest plots of the sensitivity analysis for mMRC between the FE-CB and the NE phenotypes. Figure S7. Difference of BMI between the FE-CB and the NE phenotypes. Forest plots of the difference of BMI between the FE-CB and the NE phenotypes. Figure S8. Sensitivity analysis of BMI between the FE-CB and the NE phenotypes. Forest plots of the sensitivity analysis of BMI between the FE-CB and the NE phenotypes. Figure S9. Difference of BODEx between the FE-CB and the NE phenotypes. Forest plots of the difference of BODEx between the FE-CB and the NE phenotypes. Figure S10. Difference of Charlson comorbidity index between the FE-CB and the NE phenotypes. Forest plots of the difference of Charlson comorbidity index between the FE-CB and the NE phenotypes. Flow Diagram. PRISMA 2009 Flow Diagram. The screening procedure the study. Table S1. Excluded list. List of excluded full-text articles. Table S2. Other indices. Other indices in different phenotyp. Text S1. Litera [file 12890_2020_1126_MOESM1_ESM.zip › Flow DiagramR4.doc]

**PRISMA 2009 Flow Diagram**

Records identified through database search

(n =372)

**Screening**

**Included**

**Eligibility**

**Identification**

Additional records identified through other sources

(n =0)

Records after removal of duplicates

(n =268)

Records screened

(n =36)

Records excluded

(n =19)

Full-text articles assessed for eligibility

(n = 17)

Full-text articles excluded, with reasons (n =7);

control settings are unreasonable (n=1)

No Relevant outcome indicators (n=1)

Unable to extract data (n=5)

Studies included in qualitative synthesis

(n =10)

Studies included in quantitative synthesis (meta-analysis)

(n =10)
